# The Associations of Vitamin D Status with Athletic Performance and Blood-borne Markers in Adolescent Athletes: A Cross-Sectional Study

**DOI:** 10.3390/ijerph16183422

**Published:** 2019-09-14

**Authors:** Myong-Won Seo, Jong Kook Song, Hyun Chul Jung, Sung-Woo Kim, Jung-Hyun Kim, Jung-Min Lee

**Affiliations:** 1 Department of Taekwondo, College of Physical Education, Kyung Hee University (Global campus), 1732 Deokyoungdaero, Giheung-gu, Yongin-si, Gyeonggi-do 17014, Korea; 2 Department of Kinesiology, College of Communication and Education, California State University-Chico, 400 West First Street, Chico, CA 95922, USA; 3 Department of Sports Medecine, College of Physical Education, Kyung Hee University (Global campus), 1732 Deokyoungdaero, Giheung-gu, Yongin-si, Gyeonggi-do 17014, Korea; 4 Department of Physical Education, College of Physical Education, Kyung Hee University (Global campus), 1732 Deokyoungdaero, Giheung-gu, Yongin-si, Gyeonggi-do 17014, Korea

**Keywords:** 25-hydroxivitamin D, exercise performance, stress-to-recovery status, adolescent athletes

## Abstract

The purpose of this study was to examine the associations of vitamin D status with athletic performance and blood-borne markers in adolescent athletes. This cross-sectional study included forty-seven Taekwondo athletes, aged 15–18 years old. Athletic performance was assessed using maximal oxygen consumption (VO_2max_), Wingate anaerobic power test, vertical jump, agility T-test, lower limb muscle strength, and fatigue resistance. Blood samples were collected to assess serum 25-hydroxyvitamin D [25(OH)D], free-testosterone, cortisol, creatine kinase, and urea. One-way ANOVAs were applied using Bonferroni adjusted alpha levels, which was 0.02 (i.e., 0.05/3). Multiple linear regressions analyses as well as Pearson and partial correlation analyses were used to examine the relationship among 25(OH)D concentration, athletic performance, and blood-borne markers. The participants 25(OH)D concentration were ranged from 16 to 73.25 nmol/L, indicating that 74.5% of the adolescent athletes have vitamin D insufficiency or deficiency. The vitamin D status did not show any significant effects on the performance factors or blood-borne markers. Serum 25(OH)D concentration was positively correlated with mean power output (r = 0.359, *p* < 0.05) and relative mean power output (r = 0.325, *p* < 0.05) after adjusting for bone age, height, weight, training experience, lean body mass, and fat mass. However, 25(OH)D concentration was not associated with other performance-related factors and blood-borne markers. In addition, multiple linear regressions analyses revealed that serum 25(OH)D concentration were not significant predictors of athletic performance in adolescent athletes. In conclusion, vitamin D status is weakly correlated with anaerobic capacity; moreover, the underlying mechanisms of how vitamin D influence anaerobic performance is unclear in the present study. Nevertheless, the importance of vitamin D on health benefits should not be underestimated, especially during growth periods.

## 1. Introduction

Vitamin D has been associated with positive health benefits due to its effectiveness by playing an essential role in the human endocrine systems such as muscle metabolism, cardio-metabolic risk factor, and inflammation-related cancer [1]. To date, the effects of vitamin D are interested in a variety of researchers, clinicians, and coaches [2]. While considerable evidence supports the effects of vitamin D in research, various researchers are still attempting to find new evidence linking the effect of vitamin D on other health and performance benefits.

The associations of vitamin D status with athletic performance have been issued in recent years among strength and conditioning communities because of its important role in muscle function and injury [3,4]. A meta-analysis determined that an optimal serum 25-hydroxyvitamin D [25(OH)D] helps to maintain or improve overall performance for athletes and enhance muscle contraction (i.e., myosin power stroke) and facilitate muscle repair. It has been reported that adequate vitamin D status maybe play a vital role in muscle remodeling [4,5]. Several studies also indicated that the incidence rate of muscle injuries was high among athletes who were vitamin D insufficiency [6,7]. Nevertheless, Vitamin D deficiency or insufficiency are prevalent among athletes. Marron et al. and Hamilton et al. reported that the prevalence rates of vitamin D insufficiency among outdoor sports athletes have ranged from 68.8% to 84.0% [8,9]. Jung et al. found collegiate Taekwondo athletes were 100% vitamin D insufficiency [10]. These unfavorable trends have also found among adolescent swimmers indicating that the prevalence of vitamin D insufficiency was ranged from 66.2% to 67.1% [11,12].

The relationships between 25(OH)D concentration and athletic performance have previously been observed in many studies; however, the studies found inconsistent results between serum 25(OH)D concentration and performance-related factors in athletes [9,10,13,14]. In the last decade, numerous studies have suggested a positive correlation between vitamin D and muscle function [15,16,17,18,19]. However, no significant effects were found on athletic performance when compared to deficient, insufficient, and adequate vitamin D status [20]. Those studies mentioned earlier did not consider the individuals training status, sex, and different age groups [3,21]. Therefore, it is still nebulous whether physiological benefit on a different sports event, training experience, and effects of vitamin D to be completely elucidated in athletic performance [22].

The challenges of performance-enhancing strategies in the athlete are certainly complicated due to the athletes’ condition, nutrition intake, and training process (i.e., periodization plan). Thus, aspects of vitamin D status in the athlete have drawn attention to the adaptive training performance as well as metabolic system (i.e., muscle, bone, immune, and cardiac function). The status of training intensity or overtraining syndromes can be determined by examining blood-borne markers such as plasma free testosterone (FT), cortisol (C), creatine kinase (CK), and urea (U). It also provides precise and reliable measurements of the physiological response to identify the stress-to-recovery status. The assessment of blood-borne markers can be useful for determining an optimal training strategy on an individual basis and athletic performance levels [23,24]. However, to the best of our knowledge, vitamin D status has not extensively investigated with blood-borne markers in the athletic performance. This present study systemically examines whether vitamin D status and stress-to-recovery status influences the athletic performance across the activation of blood-borne markers. Therefore, it was hypothesized that there would be different effects between vitamin D status with athletic performance and blood-borne markers in adolescent athletes.

## 2. Materials and Methods

### 2.1. Participants

Forty-seven male adolescent Taekwondo athletes (age: 16.7 ± 0.84 years, height: 175.2 ± 5.97 cm, weight: 66.2 ± 10.46 kg, and training experience: 53.4 ± 10.60 months) volunteered to participate in the study. All participant regularly performs physical training five times a week, at least three hours per day. Inclusion criteria to participate in the study were; (1) over three years of Taekwondo experiences, (2) no history of medication and disease, (3) no skeletomuscular injury experience in the last six months, and (4) taking vitamin D supplement within last three years. The study procedure including study design, benefit, and possible risks were fully explained to participants and their parents, and a written consent form approved by the Institutional Review Board of Kyung Hee University was obtained from each participant and parent. This study was conducted during the in-season period in November at the Gyeonggi-do state, Republic of Korea (37° 15′ N).

### 2.2. Physique and Skeletal Maturation

Participants standing height and body weight were measured without shoes wearing light clothing, to the nearest 0.1 cm using a stadiometer (T.K.K. Takei Scientific Ins Co., Tokyo, Japan) and balance beam scale (Seca 841, Gmbh and Co. KG, Hamburg., Germany) to the nearest 0.1 kg, respectively. The skeletal maturity of the participants was assessed in portable x-ray scans (CORUS, Y. Cm, Growth Well Co, Korea) of the left hand, wrist, and fingers, which is 56 kV at 1.5 mmAl for radiation. Tanner-Whitehouse 3rd (TW3) edition system was used to estimate the skeletal maturity states of the 13 short or long bones (i.e., radius, ulna, metacarpal 1–3–5, proximal phalanges 1–3–5, and middle phalanges 3–5, distal phalanges 1–3–5). TW3 methods for bone age determination was calculated in the radius-ulna-short bones scores {0 (invisible) to 1000 (full maturity)} [25]. The ICC of bone age was 0.99.

### 2.3. Body Composition and Bone Mineral Density

Body composition parameters (i.e., % body fat, lean body mass, fat mass), and bone mineral density (BMD) were estimated by dual X-ray absorptiometry (DEXA: QDR-4500W, Hologic, Marlborough, MA, USA). BMD was assessed in the whole body, lumbar (L1–L4), the femur of the left, and forearm area of the left and calculated as g·cm^2^. All athletes were measured while wearing clothing, barefoot, and after removing all metal from their body for a whole-body scan and three sites of BMD area. The coefficient of variance of scanning was 1.5% or less, which was in approval with that indicated by the manufacturer. All scans were conducted by the same technician, and the intra-class correlation coefficient (ICC) of DEXA measurement in our laboratory was 0.99 [26].

### 2.4. Serum 25-hydroxyvitamin D and Blood-borne Markers

Participants were instructed to overnight fasting at least 12 h and were prohibited from any severe physical activity for 24 h prior to the blood collection. When athletes arrived in the laboratory, they sat on the chair for 30 min. Then fasting serum samples (3 mL) were taken from the antecubital vein area of the arm and collected into the tube. The collected blood samples were clotted for 30 min at room temperature (20–22 °C), and centrifuged at 3000 RPM (Revolutions per minute) for 15 min. The separated serum samples were stored at − 80 °C.

An automatic CMIA (Chemiluminescence microparticle immunoassay analyzer, Architect i2000SR, Abbott, Singapore) with serum 25(OH)D kit (ARCHITECT 25-OH Vitamin D, Abbott, Singapore) was used to assess serum 25(OH)D. Blood-borne marker including FT, C, CK, and U was analyzed. FT was analyzed by an automatic radioimmunoassay analyzer system (R counter, Packard, Meriden, USA) with free testosterone RIA CT kit (Asbach Medical Products, Obrigheim, Germany). C was determined using an automatic Electro-chemiluminescence Immunoassay (ECLIA) analyzer (Cobas 8000, Roche Diagnostics, Mannheim, Germany) with Cortisol II kit (Roche Diagnostics, Mannheim, Germany). CK was analyzed with a creatine kinase kit (Roche Diagnostics, Mannheim, Germany) and measured on a Roche Cobas 8000 Modular Analyzer (Roche Diagnostics, Mannheim Germany), which is an ultraviolet (UV) assay. U was analyzed with a UREAL kit (Roche Diagnostics, Mannheim Germany), which is a kinetic ultraviolet assay. The blood analyses were performed at the standard research laboratory (Green Cross Lab Cell, certified by Korea laboratory accreditation scheme, South Korea) and the assessment of 25(OH)D received a certificate of proficiency issued by the Vitamin D External Quality Assessment Scheme in the United Kingdom. Vitamin D status was defined as a serum 25(OH)D concentration illustrated by Institute of Medicine propose building standard criteria as status (25(OH)D < 30 nmol/L: deficient, 30–50 nmol/L: insufficient, above 50 nmol/L: Adequate) [27]. The inter- and intra-coefficient of variance were 3.6% and 1.9% for 25(OH)D, 4.8% and 4.0% for FT, 1.5% and 1.0% for C, 0.6% and 0.6% for CK, and 0.7% and 0.2% for U, respectively.

### 2.5. Athletic Performance

Athletic performance measurements included aerobic capacity (i.e., Graded maximal exercise test), anaerobic capacity (i.e., Wingate anaerobic power test), power (i.e., Vertical jump), agility (i.e., Agility T-test), lower limb muscle strength (i.e., Isokinetic knee muscle strength), and fatigue resistance (i.e., Eccentric contraction fatigue protocol) on laboratory and field tests.

A graded maximal exercise test using the Bruce protocol was applied on a motor-driven treadmill (Series 2000, Marquette Electronics, Wisconsin, USA) to measure participant aerobic capacity. After 5 min of warm-up exercise, participants wore the mask connected with the metabolic cart (Quark B^2^, Cosmed, Rome, Italy) and sat on the chair till the respiratory exchange ratio (RER) level dropped to 0.8. The test ceased when participants VO_2_ and heart rate do not show the corresponding responses with increasing the speed and incline, the rating perceived exertion (RPE) exceeded 17 on the Borg scale, over 90% of their age-predicted maximum heart rate and the respiratory exchange ratio (RER) exceeded 1.15 [28]. Maximal oxygen uptake (VO_2max_) was recorded, and the ICC of the VO_2max_ assessed was 0.95 [29].

The Wingate anaerobic power test (WAPT) was used to estimate the anaerobic capacity. Participants were instructed to cycling for 2–4 min at a resistance of 0.5 kilopond, maintaining 60 RPM as warm-up protocol. Then, participants performed maximal cycling against a resistance pre-decided resistance kilopond (0.075 _kp_/body weight) for 30 s on a cycle ergometer (Monark 864, Monark-Crescent AB, Varberg, Sweden). Peak power, relative peak power, mean power, and relative mean power output were calculated using the equation {Power output (kpm × min^−1^) = [revs × resistance (kg) × distance (m) × 60 (sec)]/time (sec), Watts = kpm × min^−1^/6.123, Watts/kg = Watts/body weight (kg)} [30]. Verbal encouragement was applied to maximize athletes’ performance. The reliability of repeated measurements estimated by the ICC was 0.96 [29].

Power test was performed on a vertical jump. Participants were instructed to stand on the circular rubber mat (38 cm in diameter) to a jump measurement device (Jump-MD, Takei, Tokyo, Japan) with tightened the testing belt around their waist. The participant jumped vertically with both lower limbs as high as possible using a counter movement. The best score of two trials was recorded the 1 cm with 5 min of rest between trials, and the ICC of vertical jump was 0.97 [29].

The agility T-test was used to assess speed with multi-directional movement (i.e., forward, lateral, and backward). Participants were asked to be ready at the starting line con A. After a verbal signal (i.e., “ready, go”), they performed maximal sprint to cone B and touched the top of the cone B with the right hand. Then, shuffled as quickly as possible with side-steps to con C and touched the top of the con C with their left hand. Participants then shuffled to the reverse direction (i.e., right) using side-steps to cone D and touched the top of the cone D with their right hand. Then, shuffled back to the reverse direction (i.e., left) and touched its top of the cone B. At last, the athletes were asked to sprint backward maximally and returned to con A. The stopwatch (HS-3, Casio, Tokyo, Japan) was recorded from the start to the end of the ability T-test. The lowest score of two trials was recorded to the 0.01 s, and the reliability of the repeated measurements (ICC) of the agility T-test in our laboratory lab was 0.96 [29].

Lower limb muscle strength was measured by assessing isokinetic muscle of the quadriceps (knee extensor) and hamstrings (knee flexor) with an isokinetic dynamometer (Cybex Humac Norm Model 770, Computer Sports Medicine Inc., New York, NY, USA). Participants performed a full range of motion for peak torque with, five maximal effort contractions at 60°/sec. The data were normalized with each participants’ body weight and calculated as peak torque [(Nm) ÷ (kg)]. The reliability of the repeated measurements assessed by the ICCs was ranged from 0.97 [10].

Fatigue resistance was measured using an isokinetic dynamometer (Cybex Humac Norm Model 770, Computer Sports Medicine Inc., NY, USA). The participant performed three sets of twenty maximal extension contractions at 120°/sec with 1 min intervals, and the researcher provided verbal feedback of encouragement. Only the dominant leg of preference was assessed. The total work done was calculated as the sum of work done during the three sets, and each set was recorded. Fatigability resistance was calculated using the % of the knee extension relative values achieved in the 3^rd^ set per the 1^st^ (i.e., FR = (3^rd^ set/1^st^ set) × 100). Rating perceived exertion (Borg scale; 17.0 ± 1.16) and heart rate (Polar RS400, Polar Electro OY, Kemple, Finland) were recorded at the pre-and post-tests of each set (Peak heart rate; 155 ± 14.05 bpm). Blood samples for lactate concentration (mmol/L) were acquired 4 times from fingertip with strips (Accutrend^®^ lactate, Roche Diagnostics, Mannheim, Germany) by Accutrend^®^ Plus (Roche Diagnostics, Mannheim Germany), before the test, immediately after the test, and 5 and 10 min after the rest (peak lactate; 8.9 mmol/L). The reliability (ICC) of the isokinetic dynamometer ranged from 0.92 to 0.93 for the total work done for the extensors and flexors.

### 2.6. Nutritional Intake

Nutritional intake was investigated using 3-day dietary records (two weeks days and once on the weekend) to calculate the amount of dietary energy. All the data were analyzed by using the statistical software program (CAN Pro 4.0, Korean Nutrition Society, Seoul, Korea) to assess the total energy, carbohydrate, lipid, protein, and vitamin D supplemental via food intake.

### 2.7. Statistical Analysis

Statistical analysis was performed by the SPSS software program (version 25, SPSS Inc, Chicago, IL, USA). The descriptive data were expressed as mean, standard deviations, 95% confidence interval, and effects sizes. A one-way ANOVAs were applied using Bonferroni adjusted alpha levels, which was 0.02 (i.e., 0.05/3). Pearson’s product moment correlation coefficients analysis was used to analyze the association between serum 25(OH)D, athletic performance, and blood-borne markers. Also, a partial correlation was used to evaluate the relationship between serum 25(OH)D, athletic performance, and blood-borne markers while adjusting for potential covariates. Multiple linear regressions used to identify independent predictor of 25(OH)D concentration after controlling for covariates. In multivariable model, we adjusted for skeletal maturation (bone age), height, weight, training experience, lean body mass, and fat mass. Effect sizes were calculated as partial eta-squared (η^2^_p_; small ≥ 0.01, medium ≥ 0.06, large ≥ 0.14) values within measures ANOVA. The intra-class correlations (ICC) were calculated to evaluate the test-retest reliability for variables, with an ICC of representing poor (<0.5), moderate (0.5–0.75), good (0.75–0.9), and excellent (>0.9). The statistical significance level was set at 0.05.

## 3. Results

Descriptive statistics and group difference among participants’ characteristics with vitamin D status are shown in Table 1. The serum 25(OH)D concentration ranged from 16 to 73.25 nmol/L; with 74.5% of the adolescent Taekwondo athletes owing to vitamin D status deficiency or insufficiency. No significant difference effects were found among the vitamin D status in all athletic performance variables (Table 2). In addition, there was no significant difference between the three groups in blood-borne markers (Table 3). A significant Pearson correlation was not detected between serum 25(OH)D concentration, mean power output and relative mean power output, but this association remained after adjusting for model 2 (mean power output, r = 0.359, *p* < 0.05; relative mean power output, r = 0.325, *p* < 0.05). Also, serum 25(OH)D concentration was not connected with FT, C, CK, and U (Table 4). As with blood-borne markers, FT was correlated with mean power output (r = 0.291, *p* < 0.05), and adjusting for model 1 (r = 0.306, *p* < 0.05). However, FT was not correlated with mean power output adjusting for model 2. Model 1 and Model 2 were explained in Table 4. There were significant correlations between vertical jump (r = 0.378, *p* < 0.01), agility T-test (r = −0.381, *p* < 0.01), and FT, however no association was found after adjusting model 1 and 2. C was correlated with relative mean power output (r = −0.438, *p* < 0.01) and VO_2max_ (r = −0.317, *p* < 0.05), and after adjusting for bone age (relative mean power output; r = −0.430, *p* < 0.01, VO_2max_; r = −0.312, *p* < 0.05). However, multiple linear regressions analyses indicated that 25(OH)D concentration were not significant predictors of athletic performance in adolescent athletes (Table 5).

## 4. Discussion

The present study examined the associations of vitamin D status with athletic performance and blood-borne markers in adolescent athletes. Our findings were revealed that (a) vitamin D deficiency or insufficiency were highly prevalent among adolescent athletes (74.5%), (b) vitamin D status did not show any significant difference effects on the athletic performance factors, and (c) serum 25(OH)D concentration was weakly correlated with mean power output and relative mean power output adjusting for bone age height, weight, training experience, lean body mass, and fat mass.

Contrary to our expectations, approximately 75% of the participants were vitamin D insufficiency. Other studies also reported that the prevalence of vitamin D deficiency or insufficiency were ranged from 59% to 94% among athletes such as football players and gymnastics [7,31,32]. These inconsistent trends of vitamin D status among athletes may result in different geographical locations (i.e., latitude), races, weather conditions, and type of sports (i.e., indoor and outdoor). Although the present study and previous study were conducted with similar conditions (both Taekwondo athletes, same geographical locations, races and weather condition), the prevalence rate was lower in adolescent athletes (75%) than collegiate athletes (100%) [10]. We speculated that different sunlight exposure time may influence the serum 25(OH)D concentration between the studies. In this study, adolescent athletes participated in physical education classes (50 min/3 times per week) that was consisted of various outdoor activities such as soccer and track and field whereas collegiate athletes spent most of their training time at an indoor gym. It is believed that sunlight exposure time is the major factor for increasing 25(OH)D concentration compared to vitamin D supplement via food intake (no significantly different effects for the group in the variable) [33]. Thus, researchers recommend at least 20 min of outdoor activities to keep adequate vitamin D concentration. Nevertheless, vitamin D supplementation is still required in adolescent and appropriate optimal 25(OH)D status for indoor sports athletes.

In the present study, body composition and bone mineral density were not different among vitamin D deficiency, insufficiency, and adequate groups. Forney et al. reported that serum 25(OH)D concentration was negatively associated with and body mass index in the college students [34]. A similar result was revealed that vitamin D insufficiency had increased abdominal visceral fat, abdominal subcutaneous fat, and intramuscular fat in healthy young females [35]. Also, 25(OH)D concentration plays a role in muscle (i.e., metabolism, function) and bone turnover markers (i.e., β-CTx) [36,37]. However, the results of the study were inconsistent with previous studies. It was speculated that no difference between vitamin D status and individual characteristics might be connected with substantially to training experience and athletic performance levels in the adolescent.

Optimal vitamin D status is essential not only to prevent musculoskeletal injuries but also to improve athletic performance [3,26]. However, our findings showed that athletic performance-related factors were not different among vitamin D deficient, insufficient, and adequate groups. A recent meta-analysis pointed out that the improvement of vitamin D might be possible, as it is progressed optimal dosing regimen to improve for muscle strength in the young and healthy population [38]. Also, various studies demonstrated that correcting vitamin D deficient improves anaerobic capacity, vertical jumping height, velocity, and competition level [10,22,39]. Conversely, Farrokhyar et al. reported that correcting vitamin D level was not associated with performance improvement in the meta-analysis study [40]. The authors reported that the well-designed study, systematically controlled extraneous variables such as various sports event, environmental location, race, and diagnostic criteria, is needed to confirm the effects of vitamin D supplementation on athletic performance. Thus, future studies are required to examine the controlling of these considerations and should help to further elucidate the exact mechanism in vitamin D and athletic performance.

The present study found that vitamin D was not clearly associated with athletic performance. Similarly, Ksiazek et al. reported no correlation between 25(OH)D concentration and VO_2max_ in soccer players [41]. Conversely, Valtuena et al. found that vitamin D status is associated with VO_2max_ in male adolescent (r = 0.108, *p* < 0.05) [42]. Another previous study reported that free supplementation of 25(OH)D concentration associated the aerobic capacity (r = 0.436, *p* < 0.001), squat jump (r = 0.731, *p* < 0.001), countermovement jump (r = 0.740, *p* < 0.001), 10 m sprint (r = −0.649, *p* < 0.001), and 20 m sprint performance (r = 0.673, *p* < 0.001) [43]. Inconsistent results from the previous research showed that fatigue status and performance related variables might have effects on individual performance test scores in athletes [10,43]. Therefore, the question is raised whether those studies have utilized precise and reliable measurements or, rather, be conducted as an appropriate stress-to-recovery status for optimal athletic performances. However, it still remains uncertain of experimental evidence of a true association between vitamin D and athletic performance related factors.

The results of this study indicated that serum FT concentration was positively associated with mean power output, right knee extension peak torque, vertical jump, and agility T-test. On the other hand, a high concentration of C was negatively correlated with VO_2max_ and relative mean power output. It is well known that FT is a primary anabolic hormone and markedly increased following resistance training, which is known to contribute re-modeling of muscle tissue by enhancing myogenic protein synthesis and glycogen re-synthesis [23,44]. Conversely, cortisol, which is known as a catabolic hormone, but not completely unnecessary for muscle metabolism is markedly increased after overreaching and/or overtraining [45]. Previous studies showed that increased production of C inhibits protein synthesis and glycogenolytic enzyme activity, and thereby partially impairs muscle adaptation and athletic performance [23,46]. Nevertheless, stress-to-recovery ability greatly varies with different individuals and much influenced by several factors such as training experience and different level of competition [47].

Several limitations should be considered when evaluating the result of this analysis. A comparative lack of sample size with deficient (n = 5), insufficient (n = 30), and adequate (n = 12) vitamin D status, time points of measurement, and specific population, which limit the representativeness of our study. Future study should directly compare the measurement consequences following changes of vitamin D status based on weather conditions and various age groups in athletes. To correction for the limitation of the present study, we also suggested researchers to deliberately consider stress-to-recovery status, training status, seasonal variation, location, ethnicity, and ages by choice the periods during the training of various athletes. However, the strengths of our study included a comparative design utilizing the adolescent Taekwondo athletes to demonstrate the association between vitamin D status and athletic performance. Also, the representative measurement variables of a specific sports event were applied to advance the previous studies.

## 5. Conclusions

In conclusions, the present study showed that vitamin D status has no significant effects on athletic performance-related factors, even though the adolescent Taekwondo athletes had a higher rate of insufficient serum 25(OH)D concentration (74.5%). Some care should be taken into consideration in the interpretation of the association between athletic performance and blood-borne markers compared to serum 25(OH)D concentration, especially in male adolescent Taekwondo athletes. Yet, maintaining adequate serum 25(OH)D is a critically considerable strategy to enhance athletes’ health and performance in various sports events.

## Figures and Tables

**Table 1:** Comparison of descriptive characteristics based on the vitamin D status.

	Vitamin D Status			η^2^_p_
	Deficient	Insufficient	Adequate	
Sample (%)	5 (10.6%)	30 (63.8%)	12 (25.5%)	
25(OH)D (nmol/L) (95% CI)	24.7 ± 5.02 (19.9, 29.6)	39.3 ± 4.86 (37.3, 41.2)	63.1 ± 6.60 (60.5, 66.7)	0.85
Age (year) (95% CI)	16.2 ± 1.10 (15.9, 17.3)	16.8 ± 0.70 (16.5, 17.1)	16.4 ± 0.87 (15.9, 16.9)	0.08
Bone age (year) (95% CI)	16.4 ± 1.09 (15.5, 17.2)	16.7 ± 0.77 (16.4, 17.1)	16.2 ± 1.24 (15.6, 16.7)	0.19
Height (cm) (95% CI)	173.7 ± 5.62 (168.3, 179.1)	176.0 ± 5.19 (173.8, 178.2)	173.8 ± 7.86 (170.3, 177.3)	0.49
Weight (kg) (95% CI)	61.1 ± 5.95 (51.7, 70.6)	67.7 ± 11.72 (63.8, 71.5)	64.6 ± 10.46 (58.5, 70.7)	0.37
TE (months) (95% CI)	51.0 ± 10.75 (41.8, 60.2)	55.9 ± 10.54 (52.1, 59.7)	48.1 ± 9.20 (42.1, 54.0)	0.08
%BF (%) (95% CI)	11.4 ± 2.01 (8.2, 14.7)	13.3 ± 3.91 (12.0, 14.7)	12.6 ± 3.33 (10.5, 14.7)	0.53
LBM (kg) (95% CI)	52.0 ± 5.36 (45.4, 58.7)	55.9 ± 7.45 (53.2, 58.6)	53.9 ± 7.77 (49.6, 58.2)	0.03
FM (kg) (95% CI)	6.9 ± 1.15 (3.4, 10.4)	9.3 ± 4.61 (7.8, 10.7)	8.1 ± 1.92 (5.8, 10.3)	0.04
WBMD (g/cm^2^) (95% CI)	1.2 ± 0.09 (1.1, 1.2)	1.2 ± 0.09 (1.2, 1.3)	1.2 ± 0.10 (1.2, 1.3)	0.06
HBMD (g/cm^2^) (95% CI)	1.1 ± 0.10 (1.0, 1.1)	1.2 ± 0.11 (1.1, 1.2)	1.2 ± 0.11 (1.1, 1.3)	0.08
LBMD (g/cm^2^) (95% CI)	1.0 ± 0.13 (0.9, 1.1)	1.1 ± 0.12 (1.0, 1.1)	1.0 ± 0.15 (1.0, 1.1)	0.03
FBMD (g/cm^2^) (95% CI)	0.6 ± 0.07 (0.5, 0.6)	0.6 ± 0.06 (0.6, 0.6)	0.6 ± 0.04 (0.6, 0.6)	0.03
Energy intake (kcal) (95% CI)	2078.9 ± 209.89 (1781.9, 2375.8)	2318.7 ± 377.79 (2197.4, 2439.9)	2298.8 ± 204.67 (2107.1, 2490.5)	0.05
Carbohydrate (g) (95% CI)	303.1 ± 54.42 (267.0, 339.2)	317.7 ± 41.94 (303.0, 332.5)	307.0 ± 26.40 (283.7, 330.3)	0.02
Lipid (g) (95% CI)	60.8 ± 12.02 (45.1, 76.4)	75.3 ± 19.36 (68.9, 81.7)	78.5 ± 12.85 (68.4, 88.6)	0.08
Protein (g) (95% CI)	77.1 ± 6.09 (63.0, 91.2)	91.6 ± 18.13 (85.9, 97.4)	91.0 ± 9.85 (81.9, 100.1)	0.08
Vitamin D (IU) (95% CI)	104.0 ± 16.46 (57.6, 152.0)	132.0 ± 61.17 (113.2, 151.6)	144.0 ± 31.79 (112.0, 173.6)	0.04

Values are mean ± standard deviation. Note: TE; Training experience, %BF: % body fat, LBM: lean body mass, FM: fat mass, WBMD: whole body bone mineral density, HBMD; hip bone mineral density, LBMD: lumbar bone mineral density, FBMD: forearm bone mineral density, 95% CI; 95% confidence interval, η^2^_p_; partial eta squared.

**Table 2:** Comparison of athletic performance based on the vitamin D status.

	Vitamin D Status			η^2^_p_
	Deficient	Insufficient	Adequate	
VO_2max_ (ml·kg^-1^·min^−1^) (95% CI)	64.2 ± 1.39 (58.8, 69.6)	64.0 ± 6.94 (61.8, 66.2)	64.2 ± 3.90 (60.7, 67.7)	0.00
PPO (watts) (95% CI)	620.8 ± 75.76 (524.8, 716.9)	715.4 ± 117.85 (676.2, 754.6)	689.9 ± 81.81 (627.9, 751.9)	0.07
RPPO (W/kg) (95% CI)	10.1 ± 0.59 (9.4, 10.8)	10.6 ± 0.88 (10.3, 10.9)	10.7 ± 0.48 (10.2, 11.1)	0.04
MP (watts) (95% CI)	455.1 ± 38.93 (399.7, 510.4)	502.1 ± 60.69 (479.5, 524.6)	502.5 ± 69.37 (466.7, 538.2)	0.06
RMPO (W/kg) (95% CI)	7.5 ± 0.39 (6.9, 8.1)	7.5 ± 0.76 (7.3, 7.8)	7.8 ± 0.42 (7.4, 8.2)	0.03
REPT (Nm/kg) (95% CI)	347.0 ± 46.76 (298.8, 395.2)	327.3 ± 50.26 (307.6, 346.9)	316.6 ± 63.15 (285.5, 347.7)	0.03
RFPT (Nm/kg) (95% CI)	170.6 ± 24.68 (148.3, 192.9)	188.5 ± 22.53 (179.4, 197.6)	184.0 ± 29.98 (169.6, 198.4)	0.05
LEPT (Nm/kg) (95% CI)	347.0 ± 52.98 (309.8, 384.2)	339.0 ± 39.94 (323.8, 354.2)	324.3 ± 39.76 (300.3, 348.3)	0.03
LFPT (Nm/kg) (95% CI)	182.6 ± 40.27 (159.3, 205.9)	181.1 ± 23.98 (171.6, 190.6)	195.3 ± 23.64 (180.2, 210.3)	0.06
FR (%) (95% CI)	81.7 ± 5.61 (75.6, 87.9)	87.9 ± 7.17 (85.4, 90.4)	87.3 ± 6.34 (83.4, 91.3)	0.07
Vertical jump (cm) (95% CI)	53.8 ± 6.02 (49.5, 58.1)	52.2 ± 5.01 (50.4, 53.9)	54.0 ± 3.30 (51.2, 56.8)	0.03
Agility T-test (sec) (95% CI)	10.5 ± 0.47 (10.1, 10.8)	10.5 ± 0.40 (10.3, 10.6)	10.5 ± 0.32 (10.3, 10.7)	0.00

Values are mean ± standard deviation. Note: PPO; peak power output, RPPO; relative peak power output, MP; mean power output, RMPO; relative mean power output, REPT; right knee extension peak torque, RFPT; right knee flexor peak torque, LEPT; left knee extension peak torque, LFPT; left knee flexor peak torque, FR; fatigue resistance, 95% CI; 95% confidence interval, η^2^_p_; partial eta squared.

**Table 3:** Comparison of blood-borne markers based on the vitamin D status.

	Vitamin D Status			η^2^_p_
	Deficient	Insufficient	Adequate	
FT (pmol/L) (95% CI)	48.3 ± 15.03 (38.2, 58.5)	43.5 ± 11.40 (39.4, 47.7)	41.0 ± 9.08 (34.4, 47.5)	0.03
Cortisol (μg/dL) (95% CI)	10.7 ± 3.04 (8.8, 12.6)	8.9 ± 2.19 (8.1, 9.7)	9.1 ± 1.39 (7.9, 10.3)	0.06
CK (U/L) (95% CI)	397.8 ± 325.46 (196.8, 598.8)	375.9 ± 210.93 (293.9, 457.9)	326.1 ± 207.54 (196.4, 455.8)	0.01
Urea (mg/dL) (95% CI)	26.1 ± 7.47 (19.7, 32.5)	30.7 ± 7.72 (28.0, 33.3)	33.1 ± 5.07 (28.9, 37.2)	0.07

Values are mean ± standard deviation. Note: FT; Free testosterone, CK; Creatine Kinase, 95% CI; 95% confidence interval, η^2^_p_; partial eta squared.

**Table 4:** Pearson and partial correlation between 25(OH)D with athletic performance and blood-borne markers.

	Pearson	Partial Correlation	
		Model 1	Model 2
	r	r	r
VO_2max_ (ml·kg^-1^·min^-1^)	0.03	0.01	0.03
PPO (watts)	0.05	0.10	0.21
RPPO (W/kg)	0.13	0.12	0.20
MP (watts)	0.16	0.21	0.36 *
RMPO (W/kg)	0.21	0.12	0.33 *
REPT (Nm/kg)	−0.13	−0.06	−0.10
RFPT (Nm/kg)	0.02	0.08	0.04
LEPT (Nm/kg)	−0.10	−0.04	0.06
LFPT (Nm/kg)	0.13	0.14	0.09
FR (%)	0.13	0.12	0.24
Vertical jump (cm)	0.15	0.21	0.21
Agility T-test (sec)	−0.03	−0.13	−0.11
Free testosterone (pmol/L)	−0.19	−0.10	−0.16
Cortisol (μg/dL)	−0.06	−0.04	−0.03
Creatine Kinase (U/L)	−0.05	−0.08	−0.11
Urea (mg/dL)	0.27	0.28	0.30

Note: Model 1; adjusted for bone age, Model 2; adjusted for bone age, height, weight, training experience, lean body mass, fat mass; PPO: peak power output, RPPO: relative peak power output, MP: mean power output, RMPO: relative mean power output, REPT: right knee extension peak torque, RFPT: right knee flexor peak torque, LEPT: left knee extension peak torque, LFPT: left knee flexor peak torque, FR: fatigue resistance; * Statistically significant at *p* < 0.05.

**Table 5:** Association of athletic performance with 25(OH)D by multiple regression analyses.

				Model 1			Model 2		
	*β*	95% CI	ΔR^2^	*β*	95% CI	ΔR^2^	*β*	95% CI	ΔR^2^
VO_2max_ (ml∙kg^−1^∙min^−1^)	0.03	−0.12, 0.14	0.00	0.01	−0.13, 0.14	0.00	−0.08	−0.08, 0.10	0.00
PPO (watts)	0.05	−1.98, 2.82	0.00	0.10	−1.65, 3.19	0.01	0.09	−0.35, 1.71	0.01
RPPO (W/kg)	0.13	−0.01, 0.03	0.02	0.12	–0.01, 0.03	0.01	0.16	0.01, 0.02	0.02
MP (watts)	0.16	−0.61, 2.10	0.03	0.21	−0.41, 2.32	0.04	0.18	0.13, 1.54	0.03
RMPO (W/kg)	0.21	0.00, 0.03	0.05	0.20	−0.01, 0.02	0.04	0.23	0.00, 0.02	0.05
REPT (Nm/kg)	−0.13	−1.67, 0.08	0.02	−0.06	−1.34, 0.90	0.00	−0.08	−1.37, 0.72	0.01
RFPT (Nm/kg)	0.02	−0.52, 0.58	0.00	0.08	−0.39, 0.69	0.01	0.03	−0.44, 0.56	0.00
LEPT (Nm/kg)	−0.10	−1.20, 0.61	0.01	−0.03	−0.99, 0.79	0.00	−0.06	−1.07, 0.72	0.00
LFPT (Nm/kg)	0.13	−0.33, 0.82	0.02	0.15	−0.31, 0.87	0.02	0.08	−0.42, 0.73	0.01
FR (%)	0.13	−0.09, 0.22	0.02	0.12	−0.10, 0.22	0.01	0.23	−0.04, 0.27	0.05
Vertical jump (cm)	0.14	−0.05, 0.15	0.02	0.21	−0.03, 0.17	0.04	0.20	−0.04, 0.17	0.04
Agility T-test (sec)	−0.03	−0.01, 0.01	0.00	−0.12	−0.01, 0.00	0.01	−0.10	−0.01, 0.01	0.01

Note: Model 1; adjusted for bone age, Model 2; adjusted for bone age, height, weight, training experience, lean body mass, fat mass; PPO; peak power output, RPPO; relative peak power output, MP; mean power output, RMPO; relative mean power output, REPT; right knee extension peak torque, RFPT; right knee flexor peak torque, LEPT; left knee extension peak torque, LFPT; left knee flexor peak torque, FR; fatigue resistance, 95% CI; 95% confidence interval.
